# Phylogeographic Investigation of an Endangered Longhorn Beetle, *Callipogon relictus* (Coleoptera: Cerambycidae), in Northeast Asia: Implications for Future Restoration in Korea

**DOI:** 10.3390/insects12060555

**Published:** 2021-06-15

**Authors:** Ji Hyoun Kang, Dae-Am Yi, Alexander V. Kuprin, Changdo Han, Yeon Jae Bae

**Affiliations:** 1 Korean Entomological Institute, Korea University, Seoul 02841, Korea; jihyounkang@korea.ac.kr; 2 Research Center of Natural Monument Insects, Yeongwol Insect Museum, Yeongwol 26210, Korea; 2bigstone@korea.ac.kr; 3 Federal Scientific Center of the East Asia Terrestrial Biodiversity, Far Eastern Branch of the Russian Academy of Sciences, 690022 Vladivostok, Russia; kyprins@mail.ru; 4 Wildlife Research Center, Korea University, Ogawa-chô, Kodaira City, Tokyo 187-0032, Japan; changdo0429@hotmail.co.jp; 5 Division of Environmental Science and Ecological Engineering, Korea University, Seoul 02841, Korea

**Keywords:** *COI*, *COII*, endangered species, longhorn beetle, restoration, saproxylic insect

## Abstract

**Simple Summary:**

The critically endangered *Callipogon relictus* is one of the largest longhorn beetle species and also the only remnant species of the genus *Callipogon* found in the Palearctic region. Knowledge of the phylogeographic history and current conservational status is essential to understand its unique distribution and to establish efficient conservation strategies. We studied the phylogeographical pattern and genetic diversity of *C. relictus* from almost its entire geographical range. The level of genetic diversity and divergence of *C. relictus* from the known distributions in four regions in Russia, China, North Korea, and South Korea using two mitochondrial markers (*COI* and *COII*) revealed that genetic diversity lies within the range of intraspecific levels. Relatively high genetic diversity with no distinct phylogeographic structures indicates that the wide range of current fragmented populations might be the remnant of genetically diverse populations in the past. The results of our phylogenetic and population genetic analyses suggest that *C. relictus* populations surveyed in the present study can be used as a “source” for a future restoration and conservation program. The findings of the genetic characteristics and phylogeographic history of *C. relictus* will help to establish effective conservation strategies for this endangered longhorn beetle species in Northeast Asia.

**Abstract:**

The longhorn beetle, *Callipogon* (*Eoxenus*) *relictus* Semenov, is the only remnant species found in the Palearctic region, while all other *Callipogon* species are distributed mainly in Central America and partly in South America. This species has been placed in the first category (as ‘critically endangered’) of the Red Data Book in Russia and designated as one of the top-priority target species among all endangered invertebrate species for restoration in South Korea since 2006. Although its restricted distribution in Northeast Asia with a high conservational value has been highlighted, genetic features of *C. relictus* from different geographic regions remain unexplored. We first investigated the level of genetic diversity and phylogeographic patterns of *C. relictus* to evaluate the current conservational status and the feasibility of the implementation of a restoration program. The average genetic divergence of mitochondrial gene *COI* based on Kimura-2-parameter distance among the four regions in Russia, China, North Korea, and South Korea was 2.2%, which lies within the range of intraspecific levels. However, two separate clades with 3.8% divergence were identified, despite no geographical clustering of haplotypes. The linear pattern of the haplotype network with a high level of haplotype and nucleotide diversities suggests that the wide range of currently fragmented populations might be the remnant of genetically diverse populations in the past. This study will provide crucial information on the genetic characteristics and phylogeographic history of *C. relictus*, which will help to establish conservation strategies for this cherished insect species in Northeast Asia.

## 1. Introduction

**Figure 1:**
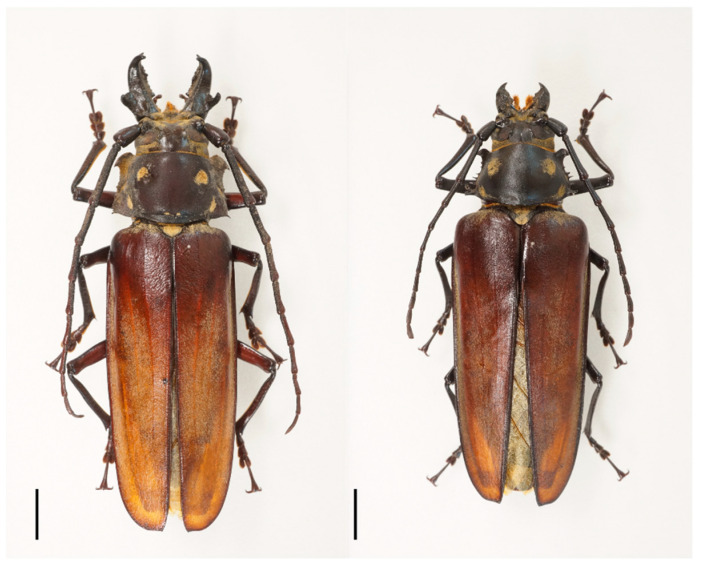
Habitus of *Callipogon relictus*, male (**left**) and female (**right**) (scale 10 mm).

**Figure 2:**
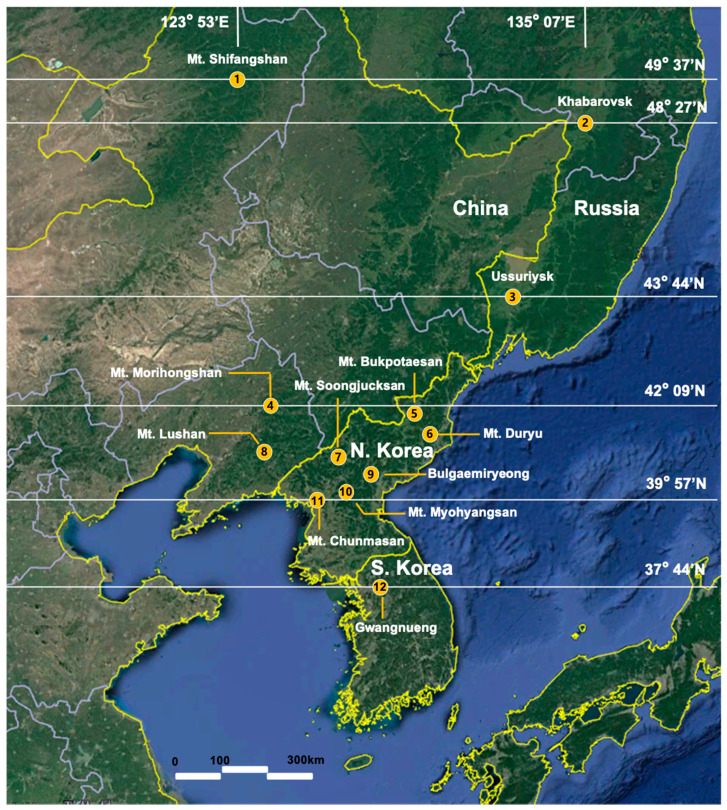
Sampling sites of *C. relictus* in the present study [11].

The critically endangered *Callipogon relictus* is one of the largest, most charismatic longhorn beetle species and also the only remnant species of genus *Callipogon* Audinet-Serville, 1832, found in the Palearctic entomo-fauna (Figure 1). Other *Callipogon* species are distributed mainly in Central America and partly in South America [1,2,3,4]. In Asia, *C. relictus* is found only in the Siberian part of the Palearctic realm comprising far-east Russia, northeast China, North Korea, and the northern part of South Korea [5,6,7,8]. The boundaries of the known habitats encompass a distance of approximately 1400 km north to south and (app.) 1800 km east to west, covering almost eleven degrees of latitude (from 48°37′ N to 37°44′ N) and twelve degrees of longitude (from 135°07′ E to 123°27′ E) (Figure 2). Among the four countries, in both Russia and South Korea, *C. relictus* was placed in the first category of the Red Data Book and it is protected by law [7,9,10]. In South Korea, it is not only listed as an endangered species but is also regarded as a national natural monument (no. 218); even the dead body of *C. relictus* is required to be registered under the regulation of Cultural Properties Protection Law.

*Callipogon relictus*, a saproxylic longhorn beetle, inhabits mixed and deciduous forests but not temperate coniferous forests; the beetles feed on the rotten logs of ancient or veteran broadleaf trees such as *Ulmus davidiana* var. *japonica* (Ulmaceae), *Quercus mongolica* (Fagaceae), *Quercus* *aliena, Quercus serrata, Carpinus* *laxiflora* (Betulaceae), etc. [7,10,12,13,14,15,16,17]. The wood-boring larva is a polyphagous internal feeder, which goes through a five- to seven-year developmental period inside rotten timber [15]. Until the beginning of the 1950s, there were two major populations of *C. relictus* in South Korea; one in the Gwangneung Forest in Gyeonggi-do (province) and the other in Chuncheon in Gangwon-do, which is located 100 km to the east of the Gwangneung Forest, almost in the same latitude (37° N) [14]. Unfortunately, the Chuncheon site was destroyed during the Korean War (1950–1953) and then submerged by the immense Soyang Dam built in 1967. Recently, distribution of *C. relictus* in the Gwangneung forest and a new location in Yangyang have been reported in Korea during four consecutive years from 2007 to 2017 [18].

While a single adult female was found every year from 2014 to 2017 [18], no female *C. relictus* was found for almost 20 years in Gwangneung Forest during the period of 1986–2006 [14], and it was not until 2015 that another female was observed [18]. These observations might indicate that the population size in the remaining habitat in South Korea is extremely small. Although the population in Gwangneung Forest is not extinct and its new location is continuously reported, the risk of local extinction in South Korea still remains high [17,18]. Conservation of *C. relictus*, therefore, has emerged as a critical need and it requires restoration of this species in Korea. Restoration practices, such as reintroduction, are becoming an effective and increasingly common strategy for conservation efforts, especially in the cases of dramatically decreasing populations in nature reserves [19,20]. For restoration of *C. relictus*, use of the current population in Gwangneung Forest is conceivable, given their continuous occurrences [18]. In some cases, however, reintroduction or translocation may be the only option when the extirpation of endangered populations is imminent [21]. Nevertheless, reintroductions of invertebrates have rarely been performed on local or global scales [21,22]. The vast majority of the reintroduction projects have dealt with vertebrate faunas, whereas only 3% have dealt with invertebrates [23]. Reintroduction or translocation have been performed for several endangered insect species of Orthoptera, Odonata, Lepidoptera, and Coleoptera all over the world (see Table S1) [20,21,22,24,25,26,27,28,29,30,31,32,33,34,35,36,37]. In fact, reintroduction or translocation of endangered insect species within a specific domestic region may be a relatively modest task as long as a potential release site has a decent habitat quality [38]. However, in order to introduce locally extinct species from overseas, conservation biologists should overcome biotic and abiotic problems, including genetic diversity, quarantines, parasites, and international legislation and regulation [39,40,41,42,43,44].

Genetic information, such as the genetic diversity level and phylogeographic history/structure of an endangered species, will assist in developing their effective conservation strategies [45,46]. It can be used for defining evolutionarily significant units or conservation units [47] to preserve the total diversity of endangered populations or species [48].

Furthermore, the level of intraspecific genetic variation has also been suggested to be the population’s capacity to adapt to novel environmental conditions [49,50,51]. In particular, small and isolated populations tend to be vulnerable to stochastic events such as depleted genetic diversity as a consequence of random genetic drift, which facilitates inbreeding depression and thereby local extinction [52]. Reintroduction or restoration for endangered populations unavoidably involves small numbers of individuals as founders. Restoration of the Asiatic black bear (*Ursus thibetanus*) in South Korea, which is one of the most successful cases for restoration of wild mammals, showed an originally low genetic diversity and the genetic admixture of two source populations, Russia and North Korea, in the restored population based on a combined analysis of mitochondrial and microsatellite markers [53]. Thus, the initial level of genetic diversity of source populations or individuals needs to be carefully considered for successful restoration practices. Understanding the phylogenetic relationships among source populations may be important for successful reintroduction [21]. However, genetic information on the populations of *C. relictus* from different distributions is not available and phylogeographic pattern has not been investigated, despite its unique distribution and conservation value.

Here, we first investigated the level of genetic diversity and divergence of *C. relictus* from the localities covering the whole distribution using two mitochondrial gene sequences, cytochrome oxidase I and II (*COI* and *COII*). We aimed to (1) test whether historical gene flow occurred among the current fragmented *C. relictus* populations in Northeast Asia and (2) to establish a knowledge base of the genetic diversity level and phylogeographic patterns amongst the geographic populations of *C. relictus* in Northeast Asia, in order to provide fundamental information on source populations for developing appropriate conservation strategies, including future restoration and reintroduction programs for this endangered insect species.

## 2. Materials and Methods

### 2.1. Sampling and Specimens

Thirty-two *C. relictus* specimens from Russia (*n* = 8, from sites 2 and 3), China (*n* = 7, from sites 1, 4, and 8), and North Korea (*n* = 17, from the sites 5, 6, 7, 9, 10, and 11) were collected between 2008 and 2015 (Table 1), preserved in 95% ethanol, and kept in Yeongwol Insect Museum, Yeongwol, South Korea. Sequences of *COI* and *COII* (JN093124 and MF521835, respectively) of *C. relictus* of South Korean origin available on NCBI GenBank database [54,55] were included, and three *Callipogon* species (*C. barbatum*, *C. lemoinei*, and *C. senex*) from Mexico were used as outgroup species. The collection locations for the sampled specimens are shown in Figure 2 and Table 1.

### 2.2. DNA Extraction, Polymerase Chain Reaction (PCR) Amplification, and Sequencing

Total genomic DNA was isolated from the legs of specimens using DNeasy Blood and Tissue Kit (Qiagen, Germantown, MD, USA), and PCR was performed to amplify fragments of mitochondrial *COI* (788 bp fragment) and *COII* (700 bp). PCR was performed with a total reaction volume of 20 μL using forward (C1-J-2183: 5′-CAACATTTATTTTGATTTTTTGG-3′) and reverse (TL2-N-3014: 5′-TCCAATGCACTAATCTGCCATATTA-3′) primers for *COI* and forward (TL2-J-3043: 5′-TGGCAGATTAGTGCAATGGATTTAA-3′) and reverse (TK-N-3768: 5′-ACTTGCTTTCAGTCATCTAATG-3′) primers for *COII* modified from published primers [56], using AccuPower PCR Premix (Bioneer, Seoul, Korea) with the following conditions: an initial denaturation at 94 °C for 1 min, 35 cycles of 30 s at 94 °C, 30 s at 50–52 °C, and 1–2 min at 72 °C, and a final extension step at 72 °C for 7 min. The PCR products were visualized on 1.5% agarose gel using UV light, purified enzymatically using Exonuclease I and Shrimp Alkaline Phosphatase (New England BioLabs, Ipswich, MA, USA), and sequenced by Macrogen INC Sequencing (Korea) using an ABI PRISM 3130xl Genetic Analyzer (Applied Biosystems, Foster city, CA, USA). Sequences of *COI* and *COII* of *C. relictus* obtained in the current study were deposited in GenBank under accession numbers MW173845–MW173876 and MW160689–MW160705, respectively. Sequences of *COI* for *Callipogon* *barbatum*, *C. senex* and *C. lemoinei* were also deposited under the accession numbers MW173877–MW173879, respectively.

### 2.3. Phylogenetic Analysis, Genetic Diversity, and Haplotype Network

A total of 36 sequence reads of *COI* (788 bp) of *C. relictus* (*n* = 33) and the three outgroup species (*C. barbatum*, *C. lemoinei*, and *C. senex*) were aligned using Clustal W implemented BioEdit v.7.0.1 [57] and manually edited. Genetic indices (i.e., genetic diversity values) were calculated and Tajima’s *D* and Fu’s *Fs* statistics estimated to test for neutrality using MEGA 4.0 [58] and ARLEQUIN v3.5 [59].

Bayesian inference (BI), maximum likelihood (ML), and neighbor-joining (NJ) analyses were conducted for phylogenetic reconstruction. BI and ML trees were inferred for all obtained sequences using MrBayes 3.2 [60] and PhyML v.3.0 [61], respectively. The NJ analysis was conducted using MEGA 4.0. The GTR+I+G model was selected as the best fit model for the BI and ML analyses using initial searches, according to the Akaike information criterion (AICc) with jModelTest v2.1.7 [62]. The BI analysis was conducted for 10 million generations, sampling every 100, and the first 25% were discarded as burn-in. The average standard deviation values of split frequencies for both runs were less than 0.01, indicating that the concurrent runs converged. The potential scale reduction factor (PSRF) of 1.0 verified the reliability of samples from the posterior probability distribution. Statistical support for both the ML and NJ analyses was obtained using 1000 bootstrap pseudo-replicates. NJ analysis was only performed for *COII* gene due to very low levels of overall intraspecific mean diversity (0.004) of *C. relictus* (*n* = 17).

The haplotypes of the 33 individuals of *C. relictus* were determined using NJ algorithms in DnaSP v5 [63]. The genetic diversity indices including haplotype diversity (*h*) and nucleotide diversity (*π*) were estimated using ARLEQUIN v3.5. A haplotype network was inferred using HAPSTAR v0.7 [64].

### 2.4. Geographic Population Structure

Spatial population genetic structure of *C. relictus* was accessed by a hierarchical analysis of molecular variance (AMOVA) analysis, implemented in ARLEQUIN v3.5. Each of the 12 populations (Table 1) was assigned to one of five geographical regions, i.e., Northeast (NE), Northwest (NW), Central North (CN), Central (C), and South (S). The populations were assigned into two groups according to the latitudinal degree. First groupings were separated into upper and lower regions from the latitude 40°00′ N and second groupings were separated by the latitude 43°00′ N. Total molecular variance was partitioned among groups (Fct = ‘inter-group’ genetic variation), populations within groups (Fsc = ‘intra-group’ genetic variation), and populations regardless of groupings (Fst = ‘inter-population’).

## 3. Results

### 3.1. Genetic Diversity and Phylogenetic Analyses

**Figure 3:**
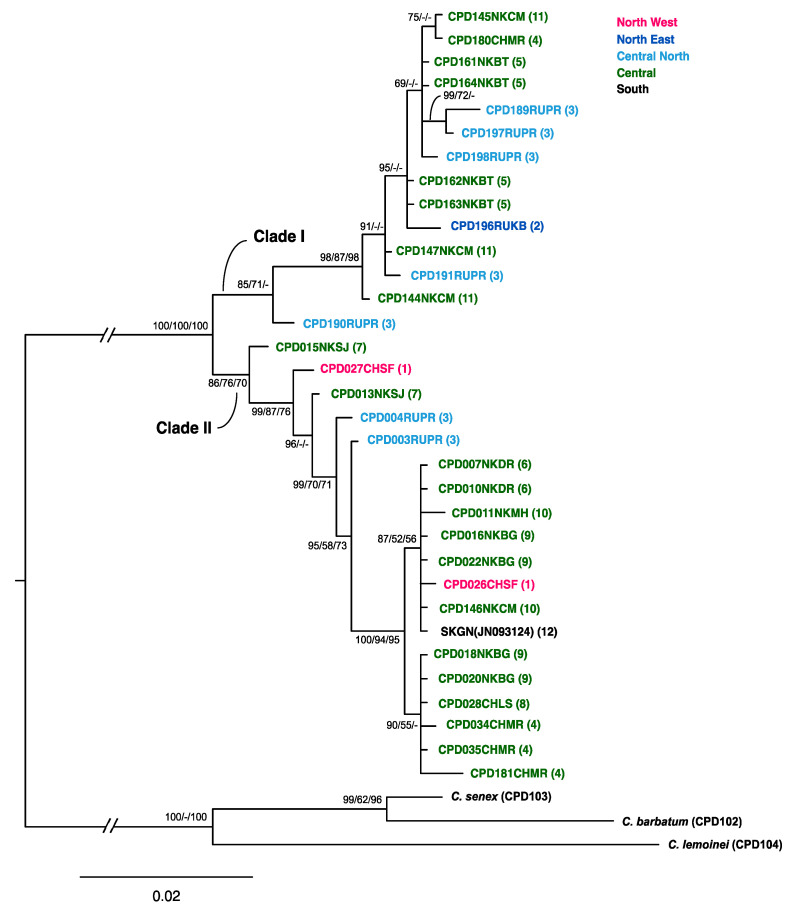
Phylogenetic tree of *C. relictus* using *COI* sequences and tree topology is shown based on Bayesian inference. Sampling site numbers are shown in the parentheses. The numbers above the nodes indicate Bayesian posterior probabilities (BI), maximum likelihood (ML), and neighbor-joining (NJ) bootstrap values, respectively. The nodes with values < 50% are shown as “-”.

**Figure 4:**
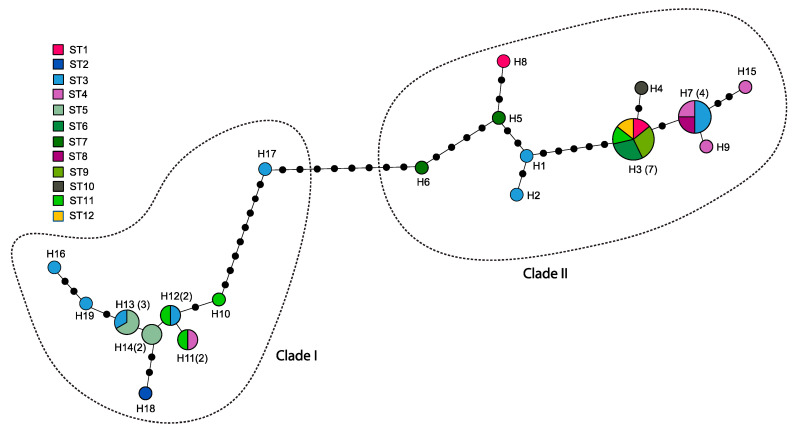
Haplotype network of *COI* sequences from *C. relictus* specimens from 12 localities. Each line represents a single mutational step, irrespective of length. Individual numbers of the respective haplotypes are presented in parentheses unless it was one individual.

A total of 17.0 and 23.7% of the sites in the *COI* alignment were variable and parsimony informative, respectively. The average genetic divergence, based on Kimura-2-parameter distance estimation, was 0.022 (ranging from 0 to 0.047) among the *C. relictus* specimens, but 0.049 (ranging from 0 to 0.219) among *C. barbatum*, *C. lemoinei*, and *C. senex* (Table S2). All *C. relictus* specimens from the 12 localities formed a well-supported monophyly, with high posterior probability (100) and bootstrap (100) values (Figure 3). Two internal clades were identified and mean distance between two clades (Clades I and II) was 0.038, with a range of 0.014 to 0.046. Mean distances within each clade were 0.005 and 0.007 for clades I and II, respectively, indicating that the genetic divergence of the geographically distant *C. relictus* individuals in each clade is low (Figure 3). Despite forming two distinct clades in all analyses (BI, ML, and NJ), no geographical clusters based on the localities were identified (Figure 3 and Figure 4). Furthermore, one specimen from South Korea (JN093124) was clustered with specimens from North Korea (DR, BG, MH: specimen identifier, Figure 3) and China (LS, MR) with high supporting values (94–100). Populations of the Shifangshan locality (Inner Mongolia, China; site 1) were genetically closer to the population of the geographically distant (> 1400 km) Gwangneung locality (South Korea; site 12) than to populations of the much closer (900–1300 km) Russian localities (KB, PR; site 3). Populations of the North Korean localities (CM, BT, SJ, DR, BG; Central region) exhibited the greatest genetic diversity and were genetically equidistant to the South Korean population (JN093124).

A total of 17 *COII* gene sequences (700 bp each) of *C. relictus* had only five parsimonious informative sites (0.7%), the genetic distance between pairwise comparisons of all the sequences based the Kimura-2-parameter model ranged from 0 to 0.012, and the overall average distance was 0.004 (Table S3). Therefore, the *COII* gene phylogenetic tree could not provide enough resolution for the phylogenetic inference, as a result of a very low level of divergence within species and among individuals from different regions, including Russia and China (Figure S1). Based on our results of *COI* and *COII* genes, we inferred that *C. relictus* specimens from Russia, China, North Korea, and South Korea might belong to a single species with identical monophyletic clustering. No distinct phylogeographic structures were detected (Figure 3 and Figure 4). However, two well-separated genetic clades of moderate levels of divergence (3.8%) in the *COI* phylogeny might indicate these may represent cryptic species diversity in *C. relictus*.

### 3.2. Haplotype Analysis

A total of 19 haplotypes of *COI* genes of specimens from the 12 localities (Figure 4) were obtained with a haplotype diversity (*h*) of 0.9375. The number of unique haplotypes (singletons) obtained from single individuals was 13 (39%), and the most common haplotype (H3, *n* = 7) was shared among North Korea (*n* = 5), China (*n* = 1), and South Korea (*n* = 1). A linear pattern of haplotype network was identified, and haplotypes, shared by more than two individuals, were located at both marginal ends of the network rather than being in the middle.

### 3.3. Geographical Population Structure and Demographic History

AMOVA could not detect any significant genetic structures between two groups when the 12 populations were divided by the latitudinal degree of 40°00′ N (I) or 43°00′ N (II) (Table 2). Molecular variances for both groupings (I and II) were −7.53 (*φ_CT_* = −0.075) and −25.85% (*φ_CT_* = −0.259), respectively. Most of the variances were observed among populations within groups and within populations in both biogeographical groupings (Table 2). Non-significant genetic structure between two groups would indicate that the historical gene flow occurred among *C. relictus* populations in Northeast Asia. All of the 12 populations were pooled as a single data set, with Tajima’s *D* and Fu’s *Fs* values of −0.83410 (*p* = 0.168) and 1.23247 (*p* = 0.707), respectively.

## 4. Discussion

We first document the phylogeographic patterns of *Callipogon relictus* remnant populations in Northeast Asia to provide baseline information for developing effective conservation or restoration strategies. *Callipogon relictus* specimens from the 12 different geographical locations, which cover almost the whole distribution of the species and extend across a distance of ~1400 km from the northern (Mt. Shifangshan, Inner Mongolia) to the southern limit (Gwangneung Forest in South Korea), did not show any distinct geographical structures (Figure 2, Figure 3 and Figure 4, Figure S1, and Table 2). We found that several of the specimens collected in Russia were more similar to those from China or even South Korea than to others from Russia (Figure 3 and Figure 4). This genetic similarity among distantly located specimens (700–800 km) and no genetic structures between two groupings by the latitudinal degree (Table 2) might explain the unique origins and evolutionary history of the species. *Callipogon relictus* has been known to have a very limited dispersal ability, as its large body size greatly impedes flight capacity [10]. In fact, individuals hardly fly and move only a few hundred meters during their 1–2-month-long adult life [10]. Therefore, gene flow by dispersal between distantly located populations (i.e., northern Russian Far East and South Korea) is unlikely, although precise estimation of direct flight ability has not been tested. Since an ongoing gene flow is unlikely owing to the poor dispersal mobility of *C. relictus,* the lack of genetic structure among populations and the linear pattern of the haplotype network with numerous mutational steps between haplotypes (Figure 4) might indicate that the current fragmented populations of *C. relictus* are remnants of genetically largely diverse ancestral populations in the past (Figure 4). Furthermore, haplotype diversity (*h*) (i.e., 0.9357) with 39% of singletons of *C. relictus* from 12 localities is much higher than other threatened saproxylic beetles. For examples, Rosalia alpina, an endangered and strictly protected saproxylic beetle, showed 0.541 of haplotype diversity from Central and South-East Europe [65] and 0.687 of haplotype diversity from the expanded regions, including a Russian population [66]. On the contrary, high levels of haplotype diversity were often identified in emerging pest species of longhorn beetles. For example, emerging or population-expanded pest species such as *Monochamus sartor*, which is one of the famous timber pests [67], *Rhagium inquisitor*, a ribbed-pine borer [68], and *Massicus raddei* [69], a serious trunk borer, showed relatively high haplotype diversity of 1.0, 0.708, and 0.96, respectively. Although a high level of genetic diversity generally indicates a large effective population size [70], the high risk of population size decreases or local extinction was reported in this species in known distributions [6,7,14,18]. This higher haplotype diversity, given the actual size of local populations, might further support the idea that a genetically diverse population of *C. relictus* existed before and fragmented into its present distributions.

Two distinct clades in the *COI* phylogeny from 12 different localities could support the hypothesis that the current regional populations of *C. relictus* that we analyzed might represent the remnants of ancestral populations with genetic mosaics. Although average genetic distance was 2.2% among all 33 individuals from 12 different sites, a much greater distance was found between several individuals belonging to the two different clades (Clade I, II; Figure 3). For example, 3.8% of genetic divergence, which was beyond the generally known intra-specific divergence level (i.e., < 2–3%) in insects [71], was identified even from individuals in the same localities. In general, > 2–3% sequence divergences have been suggested to be appropriate for delineating species boundaries in animal taxa [71], despite it surely being taxon-specific. Alternatively, two genetic forms or possibly subspecies relationships from two distinct clades are plausible (Figure 3). The haplotype network also showed that haplotypes belonging to two clades also were separated with a large number of mutational steps (Figure 4). However, individuals belonging to two distinct clades were not morphologically differentiated and geographical structures were not identified either. High intra-specific divergence of more than 4% was often observed in longhorn beetles (e.g., > 6% in *Parechthistatus gibber*) [72]. Thus, two distinct clades with high genetic divergence between several individuals could be interpreted as evidence supporting the notion that a genetically diverse population of *C. relictus* existed before and fragmented into its present distributions. However, we should note that the level of genetic diversity and phylogeographic pattern could be over- or underestimated due to the small sample size and geographical gaps of sampling sites in this study, since physical access to local populations and achieving large sample sizes for this endangered species are very challenging.

Genetic diversity in the *COI* gene is known to be a good proxy for the evaluation of the conservation status of the species in the case of a lack of previous information and census data [73]. Knowledge of the level of genetic variations and conservation status is also essential to establish a conservation plan for endangered populations, even if the sample size is smaller than ideal [74]. Although we used only a single phylogeographic marker (mtDNA *COI*) for this study, our data enable to uncover the levels of genetic diversity, divergence among populations, and also phylogeographic history of *C. relictus*. We are currently developing novel nuclear microsatellite markers, which can be further used for more detailed conservation study. The results of our phylogenetic and haplotype analyses could suggest that all the *C. relictus* sites surveyed in the present study could be used as a source population of *C.* *relictus* to the sites which are facing dramatic population decrease, as all the local populations constitute a monophyletic group with an intraspecific level of genetic divergence. For example, such reintroduction would likely improve the genetics of *C. relictus* in South Korea cases by increasing the genetic diversity of the local population via outbreeding. The present study indicates that the South Korean group exhibits the highest genetic similarity, compared to the North Korean (Central), Chinese (North West), and Russian (Central North) groups, in this order (Figure 3 and Figure 4, and Table 1). Such genetic similarity further highlights the value of the Russian and North Korean populations for conservation of this species, with a relatively large population size, in the part of the historical range where local extinction is imminent. Alternatively, if the current population in Gwangneung forest in South Korea is used for an effective conservation strategy to preserve the genetic and ecological attributes of the Korean population, which is located in the species’ southern limit (Figure 2), the level of genetic divergence can be a standard for long-term monitoring of the restored population. In both cases, the knowledge of the genetic diversity and phylogeographic pattern of *C. relictus* populations covering major geographic distributions examined in the present study would be valuable for preserving current local populations and designing the successful development of a conservation program for this endangered longhorn beetle species.

If the pattern of the current *C. relictus* population is a consequence of fragmentation from the genetically fairly varied populations in the past, the reasons behind the severe reduction in populations in South Korea, in contrast to those in Russia, should be more thoroughly studied for a successful conservation program. Dramatic decreases in the Korean peninsula population have been suggested to stem from global climate change [14]. Indeed, as *C. relictus* was found to have a limited distribution with a southernmost limit above 37° N, it may be hypothesized that the distributional range of *C. relictus* is associated with higher latitudes. The influence of temperature and global change on the abundance and distribution of terrestrial invertebrates are well-documented in most Lepidoptera [75,76,77,78,79,80] and Orthoptera [78]. However, saproxylic beetles, which are wood-boring internal feeders like most cerambycid beetles, may be less sensitive to climate change and increasing temperature than external feeders, and may even thrive under such conditions. Therefore, increased temperature could accelerate larval development [81,82], reduce the duration of diapause [83,84], and may increase the number of generations per year of multivoltine insects [81,85]. Moreover, even summer drought associated with high temperature can cause large-scale beetle outbreaks on weakened and dead host trees [85,86]. It was also reported that the ancestors of *C. relictus* were not northern or boreal species but rather adapted to the climate of Northeast Asia [4]. Recently, experimental evidence that higher temperatures both accelerate larval development and increase body size was reported [82,87]. An alternative hypothesis for the local extinction or dramatic population decreases of *C. relictus* in South Korea is that the rapid reduction in population was caused by anthropogenic habitat destruction rather than climate change. It is also worth noting that the systematic logging of *Caprinus laxiflora*, *C. laxiflora*, *Quercus aliena*, and *Q. serrata* [18], which is the principal host of *C. relictus*, in the Gwangneung Forest during the 1970s resulted in a temporal shortage of suitable host trees and dramatically reduced the Gwangneung population, which is located at the southern limit of the species distribution, in a region that may be a unique habitat in South Korea. It might be concluded that the massive logging of *C. laxiflora* colonies resulted in the simultaneous removal of multiple generations of juveniles developing in the timbers.

The information on the genetic diversity and phylogeographic pattern/structure of *C. relictus* populations, spanning almost its entire geographical range, analyzed in the present study will provide baseline, but valuable guidelines for maintaining current local populations and also for developing a successful conservation program for this endangered longhorn beetle species.

## Figures and Tables

**Table 1:** Collection and voucher information, and genetic indices of *C. relictus* samples used for DNA analysis (see Figure 2). Significant values (*p* < 0.05) are indicated in bold.

Site	Locality (Abbreviation)	GPS	Region	Specimen	*n*	NH	*h*(SD)	π (SD)	Tajima’s *D*	Fu’s *Fs*
1	Mt. Shifangshan, Inner Mongolia, China (CHSF)	49°37′ N/123°53′ E	Northwest (NW)	CPD-026 CPD-027	2	2	1.000 (0.500)	0.013 (0.014)	0	2.303
2	Khabarovsk Region, Russia (RUKB)	48°27′ N/135°05′ E	Northeast (NE)	CPD-196	1	1	1.000 (0.000)	0.000 (0.000)	0	0
3	Ussuriysk, Primoskij Kra, Russia (RUPR)	43°44′ N/131°56′ E	Central North (CN)	CPD-003 CPD-004 CPD-189 CPD-190 CPD-191 CPD-197 CPD-198	7	7	1.000 (0.000)	0.020 (0.076)	1.586	−0.9891
4	Mt. Morihongshan, Fushun, Liaoning, China (CHMR)	41°47′ N/123°58′ E	Central (C)	CPD-180 CPD-181 CPD-034 CPD-035	4	4	1.000 (0.177)	0.025 (0.017)	−0.522	1.096
5	Mt. Bukpotaesan, Ryanggang-do, North Korea (NKBT)	41°41′ N/128°18′ E	Central (C)	CPD-161 CPD-162 CPD-163 CPD-164	4	2	0.667 (0.204)	0.002 (0.002)	1.633	1.530
6	Mt. Duryusan, Hamgyeongnam-do, North Korea (NKDR)	41°08′ N/128°07′ E	Central (C)	CPD-007 CPD-010	2	1	0.000 (0.000)	0.000 (0.000)	0	0
7	Mt. Soongjucksan, Jagang-do, North Korea (NKSJ)	40°27′ N/126°23′ E	Central (C)	CPD-013 CPD-015	2	2	1.000 (0.500)	0.007 (0.007)	0	1.609
8	Mt. Lushan, Beining West, Liaonin, China (CHLS)	41°36′ N/121°42′ E	Central (C)	CPD-028	1	1	1.000 (0.000)	0.000 (0.000)	0	0
9	Bulgaemiryeong, Hamgyeongnam-do, North Korea (NKBG)	40°08′ N/127°53′ E	Central (C)	CPD-016 CPD-018 CPD-020 CPD-022	4	2	0.667 (0.204)	0.002 (0.002)	1.893	1.530
10	Mt. Myohyangsan, Pyeonganbuck-do, North Korea (NKMH)	39°57′ N/126°16′ E	Central (C)	CPD-011	1	1	1.000 (0.000)	0.000 (0.000)	0	0
11	Mt. Chunmasan, Pyeonganbuk-do, North Korea (NKCM)	40°00′ N/124°56′ E	Central (C)	CPD-144 CPD-145 CPD-146 CPD-147	4	4	1.000 (0.000)	0.024 (0.016)	−0.683	1.029
12	Gwangneung Forest South Korea	37°44′ N/127°09′ E	South (S)	JN093124 (*COI*) MF521835 (*COII*)	1	1	1.000 (0.000)	0.000 (0.000)	0	0
	Mexico (*C. barbartum*)			CPD102						
	Mexico (*C. senex*) (Outgroup)			CPD103						
	Mexico (*C. lemoinei*) (Outgroup)			CPD104						

Ussuriysk (27 m), Khabarovsk (76 m), Mt. Duryusan (2309 m), Mt. Myohyangsan (1909 m), Mt. Soongjucksan (1984 m), Bulgaemiryeong (1657 m), Mt. Shifangshan (1000 m), Mt. Chunmasan (1169 m), Mt. Bukpotaesan (2288 m), Gwangneung Forest (100–600 m).

**Table 2:** Analysis of molecular variance (AMOVA) of *C. relictus* in Northeast Asia. Significant *p*-values (*p* < 0.05) are indicated in bold.

Grouping	Source of Variation	d.f.	Sum of Squares	Variance Components	Percentage of Variation	ϕ-Statistics
2 groups (I) (Upper vs. lower from the latitude 40°00′ N)	Among groups	1	16.410	−0.66370	−7.53	ϕ_CT_ = −0.075
	Among populations Within groups	10	155.610	4.24547	48.19	ϕ_SC_ = **0.448**
	Within populations	212	109.786	5.22789	59.34	ϕ_ST_ = **0.407**
2 groups (II) (Upper vs. lower from the latitude 43°00′ N)	Among groups	1	2.679	−1.95599	−25.85	ϕ_CT_ = −0.259
	Among populations Within groups	10	169.081	4.29419	56.76	ϕ_SC_ = **0.451**
	Within populations	21	109.786	5.22789	69.10	ϕ_ST_ = **0.309**

Significant *p*-values (*p* < 0.05) are indicated in bold.

## Data Availability

Data are available upon request from the authors.

## Supplementary Materials

The following are available online at https://www.mdpi.com/article/10.3390/insects12060555/s1, Figure S1: Neighbor-joining phylogenetic tree of *C. relictus* using *COII* gene. The numbers above the nodes indicate neighbor-joining (NJ) bootstrap values. The nodes with values > 40% are shown, Table S1: Cases of reintroduction/translocation projects of endangered insect species, Table S2: Pairwise genetic distance matrix of COI among specimens of *Callipogon relictus, C. barbartum* (CPD102), *C. senex* (CPD103), and *C. lemoinei* (CPD104), Table S3: Pairwise genetic distance matrix of *COII* sequences among specimens of *Callipogon relictus* and *C. barbartum* (CPD102).
