# Supplementary material for: Phylogeographic Investigation of an Endangered Longhorn Beetle, Callipogon relictus (Coleoptera: Cerambycidae), in Northeast Asia: Implications for Future Restoration in Korea
Source: Insects. 2021 Jun 15;12(6):555. doi: 10.3390/insects12060555 (PMC8232212; doi:10.3390/insects12060555)
Supplement: Supplementary file 1 [file insects-12-00555-s001.zip › New_Table S1-3_210529.pdf]

Table 1. Cases of reintroduction/translocation projects of endangered insect species.

Order	Family	Species	From	To	References
Orthoptera	Tettigoniidae	Wartbiter cricket (*Decticus verrucivorus*)	England (Southern Egld.)	England (London Zoo)	[24]
	Acrididae	*Blue-winged grasshopper (Oedipoda caerulescens)*	Switzerland	Switzerland	[25]
		Mottled grasshopper (*Myrmeleotettix maculatus*)	England (Essex)	England (Jaywick)	[26]
Odonata (Zygoptera)	Coenagrionidae	Forktail damselfly (*Ischnura gemina*)	U.S.A. (Lion Field Park)	U.S.A. (Glen Canyon park)	[27]
		Megalagrion damselflies (*Megalagrion xanthomelas*)	Hawaii	Hawaii	[28–29]
Lepidoptera	Lycaenidae	Large blue butterfly (*Maculinea arion*)	Sweden (Öland)	England (Dartmoor etc.)	[30]
		Maculinea butterflies (*M. nausithous & M. Teleius*)	Poland	The Netherlands (Northern Brabant)	[31]
		Large copper butterfly (*Lycanea dispar batavus*)	The Netherlands	England (Woodwalton Fen)	[32]
		Karner blue butterfly (*Lycaeides melissa samuelis*)	U.S.A(Indiana)	Canada (Ontario)	[33]
	Papilionidae	Swallowtail butterfly (*Papilio machaon*)	England (Norfolk)	England (Wicken Fen)	[20]
Coleoptera	Cerambycidae	Great capricorn beetle (*Cerambyx cerdo*)	Czeck Republi (Moravia)	Czeck Republic (Hluboka)	[21]
	Cicindelidae	Tiger beetle (*Cicindela dorsalis dorsalis*)	U.S.A. (Virginia)	U.S.A. (New Jersey)	[22]
		Puritan tiger beetle (*Cicindela puritana*)	U.S.A. (Connecticut)	U.S.A. (Massachusetts)	[34]
	Silphidae	American burying beetle (*Nicrophorus americanus*)	U.S.A (Block Island)	U.S.A. (Penikese Island)	[35–36]
	Scarabaeidae	Dung beetle (*Gymnopleurus mopsus*)	Mongolia	S. Korea	[37]

Table 2. Pairwise genetic distance matrix of COI among specimens of *Callipogon relictus, C. barbartum* (CPD102), *C. senex* (CPD103), and *C. lemoinei* (CPD104).

		**1**	**2**	**3**	**4**	**5**	**6**	**7**	**8**	**9**	**10**	**11**	**12**	**13**	**14**	**15**	**16**	**17**	**18**
1	CPD003																		
2	CPD004	0.003																	
3	CPD007	0.008	0.011																
4	CPD010	0.008	0.011	0.000															
5	CPD011	0.011	0.014	0.003	0.003														
6	CPD013	0.005	0.005	0.013	0.013	0.016													
7	CPD015	0.011	0.011	0.019	0.019	0.022	0.006												
8	CPD016	0.008	0.011	0.000	0.000	0.003	0.013	0.019											
9	CPD018	0.008	0.011	0.003	0.003	0.006	0.013	0.019	0.003										
10	CPD020	0.008	0.011	0.003	0.003	0.006	0.013	0.019	0.003	0.000									
11	CPD022	0.008	0.011	0.000	0.000	0.003	0.013	0.019	0.000	0.003	0.003								
12	CPD026	0.008	0.011	0.000	0.000	0.003	0.013	0.019	0.000	0.003	0.003	0.000							
13	CPD027	0.006	0.009	0.014	0.014	0.014	0.005	0.008	0.014	0.014	0.014	0.014	0.014						
14	CPD028	0.008	0.011	0.003	0.003	0.006	0.013	0.019	0.003	0.000	0.000	0.003	0.003	0.014					
15	CPD034	0.009	0.013	0.005	0.005	0.008	0.014	0.021	0.005	0.002	0.002	0.005	0.005	0.016	0.002				
16	CPD035	0.008	0.011	0.003	0.003	0.006	0.013	0.019	0.003	0.000	0.000	0.003	0.003	0.014	0.000	0.002			
17	CPD144	0.030	0.027	0.039	0.039	0.039	0.025	0.019	0.039	0.039	0.039	0.039	0.039	0.024	0.039	0.040	0.039		
18	CPD145	0.034	0.030	0.042	0.042	0.042	0.029	0.022	0.042	0.042	0.042	0.042	0.042	0.027	0.042	0.044	0.042	0.003	
19	CPD146	0.008	0.011	0.000	0.000	0.003	0.013	0.019	0.000	0.003	0.003	0.000	0.000	0.014	0.003	0.005	0.003	0.039	0.042
20	CPD147	0.032	0.029	0.040	0.040	0.040	0.027	0.021	0.040	0.040	0.040	0.040	0.040	0.025	0.040	0.042	0.040	0.002	0.002
21	CPD161	0.035	0.032	0.044	0.044	0.044	0.030	0.024	0.044	0.044	0.044	0.044	0.044	0.029	0.044	0.045	0.044	0.005	0.002
22	CPD162	0.034	0.030	0.042	0.042	0.042	0.029	0.022	0.042	0.042	0.042	0.042	0.042	0.027	0.042	0.044	0.042	0.003	0.003
23	CPD163	0.034	0.030	0.042	0.042	0.042	0.029	0.022	0.042	0.042	0.042	0.042	0.042	0.027	0.042	0.044	0.042	0.003	0.003
24	CPD164	0.035	0.032	0.044	0.044	0.044	0.030	0.024	0.044	0.044	0.044	0.044	0.044	0.029	0.044	0.045	0.044	0.005	0.002
25	CPD180	0.034	0.030	0.042	0.042	0.042	0.029	0.022	0.042	0.042	0.042	0.042	0.042	0.027	0.042	0.044	0.042	0.003	0.000
26	CPD181	0.011	0.014	0.006	0.006	0.006	0.016	0.019	0.006	0.003	0.003	0.006	0.006	0.011	0.003	0.005	0.003	0.035	0.039
27	CPD189	0.034	0.034	0.042	0.042	0.042	0.029	0.022	0.042	0.042	0.042	0.042	0.042	0.027	0.042	0.044	0.042	0.006	0.003
28	CPD190	0.017	0.014	0.025	0.025	0.025	0.016	0.013	0.025	0.025	0.025	0.025	0.025	0.014	0.025	0.027	0.025	0.014	0.017
29	CPD191	0.032	0.029	0.040	0.040	0.040	0.027	0.021	0.040	0.040	0.040	0.040	0.040	0.025	0.040	0.042	0.040	0.002	0.002
30	CPD196	0.030	0.027	0.039	0.039	0.039	0.025	0.019	0.039	0.039	0.039	0.039	0.039	0.024	0.039	0.040	0.039	0.003	0.006
31	CPD197	0.037	0.034	0.045	0.045	0.045	0.032	0.025	0.045	0.045	0.045	0.045	0.045	0.030	0.045	0.047	0.045	0.006	0.003
32	CPD198	0.035	0.032	0.044	0.044	0.044	0.030	0.024	0.044	0.044	0.044	0.044	0.044	0.029	0.044	0.045	0.044	0.005	0.002
33	JN093124	0.008	0.011	0.000	0.000	0.003	0.013	0.019	0.000	0.003	0.003	0.000	0.000	0.014	0.003	0.005	0.003	0.039	0.042
34	CPD103	0.177	0.177	0.186	0.186	0.186	0.173	0.173	0.186	0.184	0.184	0.186	0.186	0.175	0.184	0.184	0.184	0.173	0.177
35	CPD102	0.191	0.191	0.200	0.200	0.200	0.187	0.187	0.200	0.197	0.197	0.200	0.200	0.189	0.197	0.197	0.197	0.188	0.193
36	CPD104	0.209	0.209	0.219	0.219	0.219	0.205	0.205	0.219	0.216	0.216	0.219	0.219	0.207	0.216	0.216	0.216	0.205	0.209

		19	20	21	22	23	24	25	26	27	28	29	30	31	32	33	34	35
20	CPD147	0.040																
21	CPD161	0.044	0.003															
22	CPD162	0.042	0.002	0.002														

23	CPD163	0.042	0.002	0.002	0.000													
24	CPD164	0.044	0.003	0.000	0.002	0.002												
25	CPD180	0.042	0.002	0.002	0.003	0.003	0.002											
26	CPD181	0.006	0.037	0.040	0.039	0.039	0.040	0.039										
27	CPD189	0.042	0.005	0.005	0.006	0.006	0.005	0.003	0.039									
28	CPD190	0.025	0.016	0.019	0.017	0.017	0.019	0.017	0.022	0.021								
29	CPD191	0.040	0.000	0.003	0.002	0.002	0.003	0.002	0.037	0.005	0.016							
30	CPD196	0.039	0.005	0.005	0.003	0.003	0.005	0.006	0.035	0.009	0.014	0.005						
31	CPD197	0.045	0.005	0.002	0.003	0.003	0.002	0.003	0.042	0.003	0.021	0.005	0.006					
32	CPD198	0.044	0.003	0.000	0.002	0.002	0.000	0.002	0.040	0.005	0.019	0.003	0.005	0.002				
33	JN093124	0.000	0.040	0.044	0.042	0.042	0.044	0.042	0.006	0.042	0.025	0.040	0.039	0.045	0.044			
34	CPD103	0.186	0.175	0.175	0.173	0.173	0.175	0.177	0.184	0.177	0.180	0.175	0.173	0.177	0.175	0.186		
35	CPD102	0.200	0.191	0.190	0.188	0.188	0.190	0.193	0.197	0.193	0.191	0.191	0.188	0.193	0.190	0.200	0.037	
36	CPD104	0.219	0.207	0.212	0.210	0.210	0.212	0.209	0.216	0.209	0.209	0.207	0.210	0.214	0.212	0.219	0.083	0.107

Table 3. Pairwise genetic distance matrix of *COII* sequences among specimens of *Callipogon relictus* and *C. barbartum* (CPD102).

		1	2	3	4	5	6	7	8	9	10	11	12	13	14	15	16	17
1	CPD102																	
2	CPD003	0.235																
3	CPD004	0.227	0.000															
4	CPD007	0.229	0.000	0.007														
5	CPD010	0.236	0.004	0.006	0.006													
6	CPD011	0.239	0.002	0.003	0.002	0.008												
7	CPD013	0.239	0.004	0.005	0.004	0.010	0.002											
8	CPD015	0.227	0.000	0.006	0.001	0.006	0.002	0.004										
9	CPD016	0.243	0.002	0.004	0.002	0.008	0.000	0.002	0.002									
10	CPD018	0.229	0.006	0.010	0.006	0.008	0.003	0.002	0.007	0.004								
11	CPD020	0.229	0.006	0.009	0.007	0.012	0.003	0.002	0.006	0.004	0.004							
12	CPD022	0.229	0.002	0.007	0.003	0.008	0.003	0.005	0.001	0.004	0.009	0.007						
13	CPD026	0.227	0.000	0.006	0.001	0.006	0.002	0.004	0.000	0.002	0.007	0.006	0.001					
14	CPD027	0.230	0.006	0.009	0.007	0.012	0.005	0.004	0.006	0.005	0.006	0.004	0.007	0.006				
15	CPD028	0.237	0.006	0.007	0.005	0.008	0.004	0.002	0.005	0.004	0.000	0.004	0.007	0.005	0.005			
16	CPD034	0.233	0.004	0.009	0.004	0.010	0.002	0.000	0.006	0.002	0.001	0.003	0.007	0.006	0.004	0.002		
17	CPD035	0.235	0.006	0.006	0.006	0.008	0.004	0.002	0.006	0.004	0.000	0.004	0.008	0.006	0.006	0.000	0.002	
18	SKGN_MF521835	0.235	0.002	0.009	0.004	0.008	0.000	0.002	0.003	0.000	0.007	0.006	0.004	0.003	0.007	0.004	0.006	0.004
