# Supplementary figures and images for: Phylogeographic Investigation of an Endangered Longhorn Beetle, Callipogon relictus (Coleoptera: Cerambycidae), in Northeast Asia: Implications for Future Restoration in Korea
Source: Insects. 2021 Jun 15;12(6):555. doi: 10.3390/insects12060555 (PMC8232212; doi:10.3390/insects12060555)

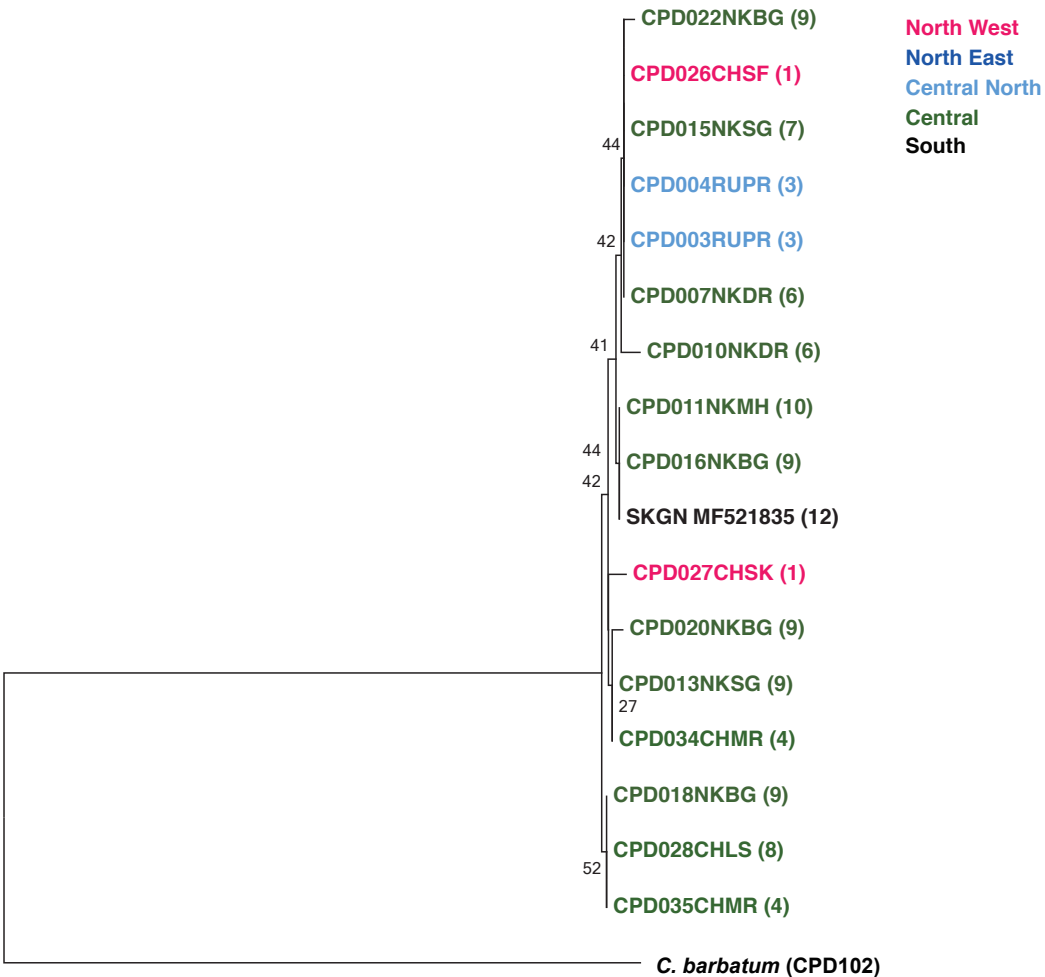

Supplement: Supplementary file 1 [file insects-12-00555-s001.zip › New_FigureS1_COII.pdf]
